# Establishing an online training program for pediatric surgery residents during and after the COVID-19 pandemic – lessons learned

**DOI:** 10.3205/zma001712

**Published:** 2024-11-15

**Authors:** Richard Gnatzy, Benjamin Schwab-Eckhardt, Annika Brunner, Sabine Drossard

**Affiliations:** 1 University Hospital Leipzig, Department of Pediatric Surgery, Leipzig, Germany; 2 Cnopfsche Children’s Hospital, Department for Pediatric Surgery and Pediatric Urology, Nuremberg, Germany; 3 Marien Hospital Witten, Department of Pediatric Surgery, Witten, Germany; 4 Ruhr-University Bochum, Medical Faculty, Chair of Pediatric Surgery, Bochum, Germany; 5 University Hospital Würzburg, Department of General, Visceral, Transplant, Vascular and Pediatric Surgery, Würzburg, Germany

**Keywords:** post-graduate education, pediatric surgery, resident training, online education, case-based learning, surgical training

## Abstract

**Aim:**

During the COVID-19 pandemic, social restrictions significantly impacted post-graduate training in pediatric surgery. This paper describes the implementation and continuation of a German-language, online training program for pediatric surgery residents, named “KiWI” (Kinderchirurgische Weiterbildung im Internet), which was established during the period of social distancing.

**Method:**

“KiWI” was designed as a monthly, post-graduate online seminar course that combined practical relevance with theoretical knowledge. The teaching methods included case presentations by residents, active participant engagement through multiple-choice questions, and chat interactions. Course evaluation was conducted via an online questionnaire. The program was specifically tailored to meet the needs of the residents through close collaboration with them.

**Results:**

Between February 2021 and September 2023, an average of 53 participants (M=53; SD=20) attended each session, with no correlation observed between attendance and the level of social restrictions. Fifty-seven percent of the participants were residents, with over half being in their fifth year or beyond of post-graduate training. Evaluations indicated a high level of satisfaction with the topics and course design. Lecturers showed great willingness to participate voluntarily, and moderators facilitated the sessions to support the lecturers. Technical issues were addressed through pre-session test runs.

**Conclusion:**

The “KiWI” project demonstrates the potential of online continuing education as a resource-efficient supplement to post-graduate medical education. The program achieved high levels of acceptance and accessibility, showcasing the effectiveness of a decentralized online seminar format with interactive, case-based teaching methods.

## Zusammenfassung

**Zielsetzung:**

Im Rahmen der COVID-19-Pandemie kam es auch in der Kinderchirurgie zu einer Einschränkung der Weiterbildung. Dieser Artikel beschreibt die Implementierung einer deutschsprachigen Online-Fortbildung für Ärzt*innen in Weiterbildung (ÄiW) unter dem Akronym „KiWI“ (Kinderchirurgische Weiterbildung im Internet) während der Kontaktbeschränkungen und die Verstetigung der Veranstaltungsreihe darüber hinaus.

**Methodik:**

Die KiWI wurde als monatliche Online-Fortbildung entwickelt und stellt neben der Vermittlung von theoretischem Wissen auch praktische Anwendungsbezüge her. Didaktische Methoden umfassen Fallpräsentationen durch ÄiW sowie aktive Einbeziehung der Teilnehmenden durch Verwendung von Multiple Choice (MC)-Fragen und Chat-Funktion. Die Evaluation erfolgte mittels eines Online-Fragebogens. Durch enge Zusammenarbeit mit den ÄiW wurde das Programm gezielt an den Bedarf angepasst.

**Ergebnisse:**

Zwischen 02/2021 und 09/2023 nahmen durchschnittlich 53 (MW=53; SD=20) Personen pro Veranstaltung teil, wobei sich kein Zusammenhang zum Ausmaß der Kontaktbeschränkungen zeigte. Siebenundfünfzig % der Teilnehmenden waren ÄiW, von denen sich über die Hälfte mindestens im 5. Weiterbildungsjahr befand. Die Evaluation zeigt eine hohe Zufriedenheit mit Themenwahl und Ausgestaltung der Veranstaltung. Es bestand eine hohe Bereitschaft der Referierenden, sich ehrenamtlich zu beteiligen. Die Moderation entlastete die Referierenden, technische Probleme konnten durch einen Probelauf vor der Veranstaltung vermieden werden.

**Schlussfolgerung:**

Das beschriebene Projekt zeigt das Potenzial von Online-Fortbildungen als ressourceneffiziente Ergänzung für die ärztliche Weiterbildung. Es zeigte sich eine hohe Akzeptanz und niederschwellige Inanspruchnahme des dezentralen Angebotes eines Online-Seminars mit interaktiven, fallbasierten Lehrmethoden.

## 1. Introduction

In recent years, online training programs and the digital transfer of scientific knowledge have gained significant importance. The COVID-19 pandemic necessitated an abrupt rethinking and development of existing concepts in medicine. The growing use of online teaching formats has transformed medical education and had already become a significant component of both undergraduate and post-graduate education even before the pandemic [1].

The COVID-19 pandemic had detrimental effects on post-graduate medical education worldwide. Due to reduced case numbers, canceled consultations and training sessions, and postponed elective surgeries, workplace-based learning opportunities for residents diminished [2], [3], including in pediatric surgery [4], [5]. Additionally, many post-graduate programs and professional conventions in pediatric surgery were either canceled or transitioned to virtual formats. In November 2020, even the most important training program for pediatric surgery residents, the Akademie für Kinderchirurgie (AKIC), was canceled.

Conversely, the pandemic also increased the proportion of theoretical training programs within post-graduate surgical training [3]. Early in the pandemic, there was a marked rise in online training offerings, despite the fact that various forms of digital learning had been established long before COVID-19 [6], [7]. Since 2020, online media has been increasingly utilized for post-graduate pediatric surgical training to disseminate current research findings and medical knowledge [8]; however, until 2021, there was no German-language program specifically aimed at pediatric surgery residents.

## 2. Project description

To address the identified needs of pediatric surgery residents, a team from the German Society for Pediatric Surgery (DGKCH) designed an online post-graduate training program named “KiWI”, which stands for Kinderchirurgische Weiterbildung im Internet (Post-Graduate Online Training in Pediatric Surgery). The development and implementation of this program are described below, following Kern’s six-step approach [9].

### 2.1. Problem identification and general needs assessment

Post-graduate surgical training in Germany typically remains unstructured, relying heavily on informal, workplace-based learning [10]. However, there is a growing demand for competency-based, structured post-graduate curricula, which are increasingly being required and implemented [11], [12]. The prevalence of shift work and rotating shifts among residents [13] often prevents them from regularly attending traditional learning opportunities, such as morning staff meetings, radiology demonstrations, ward rounds, and departmental training sessions [12]. Consequently, there is an increasing need for training sessions that are independent of time and location to accommodate residents' varied work schedules.

### 2.2. Targeted needs assessment

With 91 German hospitals authorized to provide post-graduate training in pediatric surgery [14], this represents a small target group with specific needs. The exact number of pediatric surgery residents is not precisely known, but was estimated to be 361 based on a 2017 survey of post-graduate instructors [14]. According to the national statistics of the German Medical Association (Bundesärztekammer [https://www.bundesaerztekammer.de/en/german-medical-association]), approximately 40 individuals pass the board exam in pediatric surgery each year [14]. The post-graduate curricular content for pediatric surgery is determined by the rules and regulations of each state’s medical association and covers a wide range of topics, including pediatric traumatology and pediatric urology. Depending on the structure of a given hospital, pediatric surgeons may also treat children with neurosurgical or plastic reconstructive conditions and assist in the care of children who have undergone operations in other surgical specialties, such as oral and maxillofacial surgery or pediatric orthopedics. Therefore, the specific needs of individual residents vary according to their residency location and encompass topics from many adjacent medical specialties.

The most important post-graduate training program in pediatric surgery is the “Akademie für Kinderchirurgie” (AKIC), organized annually by the DGKCH in cooperation with the pediatric professional societies in Austria (ÖGKJCH/Österreichische Gesellschaft für Kinder- und Jugendchirurgie) and Switzerland (SGKC/Schweizerische Gesellschaft für Kinderchirurgie) since 1966. This program is designed to prepare residents specifically for the board exam in pediatric surgery and is structured around four organ-based main topics: abdomen/gastrointestinal, traumatology/skeleton, urogenital tract, and head/neck/thorax/mediastinum. Since 2022, the Aesculap Akademie has also offered an annual review course in pediatric surgery as preparation for the board exam. Beyond these, there are no regularly scheduled, German-language post-graduate training programs specifically for pediatric surgery residents.

### 2.3. General and specific goals

Two main objectives were identified for the training program: first, to prepare individuals for the board exam by providing theoretical and factual knowledge; second, to support clinical work and practice by imparting clinical and practical knowledge. KiWI aims to address critical aspects of routine clinical practice that extend beyond textbook knowledge. This includes teaching expertise on topics that are either not covered or only briefly mentioned in preparation for the board exam. Additionally, the program addresses the practical management of diseases not treated at the resident’s hospital. To complement the existing program offered by AKIC, KiWI initially avoided focusing on AKIC’s main topics.

### 2.4. Educational strategies

To encourage active learning, various teaching strategies described in the literature were selected during the development of the program based on literature research [15], [16]. The course begins with case presentations by residents, who were recruited by the instructors at their respective hospitals. By establishing a clinical connection, participants are better able to integrate and remember the subsequent information [15], [17]. Following the case presentation, the lecturer delivers a lecture on the scheduled topic, based on predefined learning objectives communicated in the course invitation. Special attention is paid to the lecturers’ teaching abilities, demonstrated through their previous lectures, presentations, and continuing education, in addition to their expertise on the topic.

In selecting topics, a balance between “traditional” pediatric surgery subjects and more specific content was sought to cover the full spectrum described above. Topics in adjacent fields related to pediatric surgery were also included to provide a broader perspective on the specialty.

For technology, Zoom^®^ meetings were deliberately chosen instead of a webinar format to enable direct interaction among participants. During the intervals between the different sections of the lecture, multiple-choice questions were posed using Zoom^®^’s integrated survey tools, with attendees receiving immediate feedback. The chat function, along with the camera and/or microphone functions, were used to generate ideas and facilitate direct interaction among participants, presenters, and lecturers. Participants were actively encouraged to ask questions and discuss the material presented, addressing the different approaches taken at various hospitals. A moderator managed the chat questions and coordinated interactions among participants during each session.

### 2.5. Implementation

**Figure 1:**
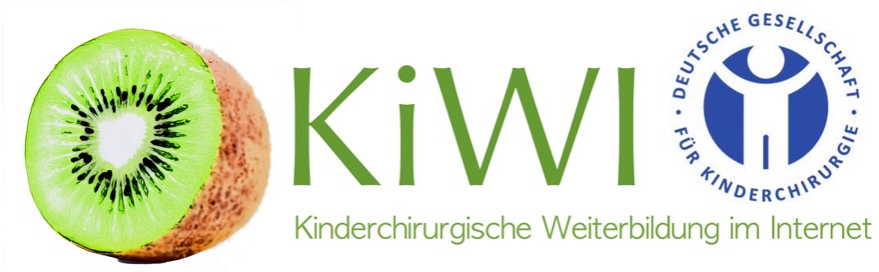
Logo for the training program

The course format was developed via videoconferences by a small team consisting of three residents and two pediatric surgeons who came together within the DGKCH. Each seminar session was set at a duration of 90 minutes, comprising 10-15 minutes for the case presentation and 30-45 minutes for discussion and questions. To establish a clear routine, the course was scheduled to be held on the last Wednesday of each month. A high degree of name recognition was achieved by using the acronym “KiWI”, designing a catchy logo (see figure 1 (Fig. 1)), and including the KiWI emoji on all of the invitations. Topics were selected by the organizational team, with participants’ suggestions and requests being actively solicited and incorporated. The organizational team met virtually one to two times per year. Existing Zoom^®^ licenses were used.

The then-president of the DGKCH supported the initial implementation of the program by acquiring the first lecturers and hosting the opening session. As the format gained recognition within the German-speaking pediatric surgery community, lecturer acquisition was transitioned to the KiWI team. In addition to individuals in leadership positions, younger specialists and senior hospital physicians were also contacted for participation. Lecturers were contacted via email or telephone in advance and briefed on the course details. Four weeks prior to the session, they received detailed information via email regarding objectives, methods, and the KiWI sequence of events. To minimize technical issues, starting from the third session onward, advance meetings were scheduled with presenters to conduct “tech checks” and address any potential problems. In individual cases (connections to hospital networks in particular), there were unexpected problems which could be successfully solved in advance. Residents on the organizational team were specifically chosen to serve as moderators and were responsible for compiling and supplementing the attendees’ questions.

Invitations were distributed via existing email distribution lists for the working group of residents in pediatric surgery, the DGKCH forum, and, from May 2023, the society’s social media channels. Initially, interested recipients were required to register, but as the program progressed, the link was made publicly accessible. From the second course in March 2021 onward, KiWI obtained certification from the Bavarian Board of Physicians (Bayerische Landesärztekammer) for two continuing education credit points. Seminar sessions were recorded in cooperation with lecturers and, with consent from participants, video podcasts were produced and made available indefinitely in the password-protected section of the DGKCH’s website for its members.

### 2.6. Evaluation

During the implementation phase from February to June 2021, an online evaluation was conducted immediately after each session to ensure quality and facilitate further development. The evaluations included questions about participants’ age, level of post-graduate education, and professional situation, along with inquiries about the range and relevance of topics, case presentations, lectures, and participants’ knowledge gain. The questionnaire was developed based on the evaluation questions used by AKIC and adapted for KiWI. Ratings were provided on a six-point Likert scale, mirroring the grading scale used in the German school system (1=excellent, 6=fail), with an option for free-text comments. The initial survey also included a question about participants’ preferred time of day.

In December 2021, an interim evaluation was conducted to assess participants’ needs and to adjust and expand sessions for the following year. Questions were posed regarding satisfaction with selected topics and desired subject areas for future sessions.

The questionnaire underwent two revisions after the pilot phase and was expanded to include inquiries about case presentations and the interactive course design to evaluate teaching methods and strategies. Since January 2022, the resulting questionnaire has been regularly used to evaluate the course. The link to the evaluation survey was provided to participants during each KiWI session via the chat.

## 3. Results

**Figure 2:**
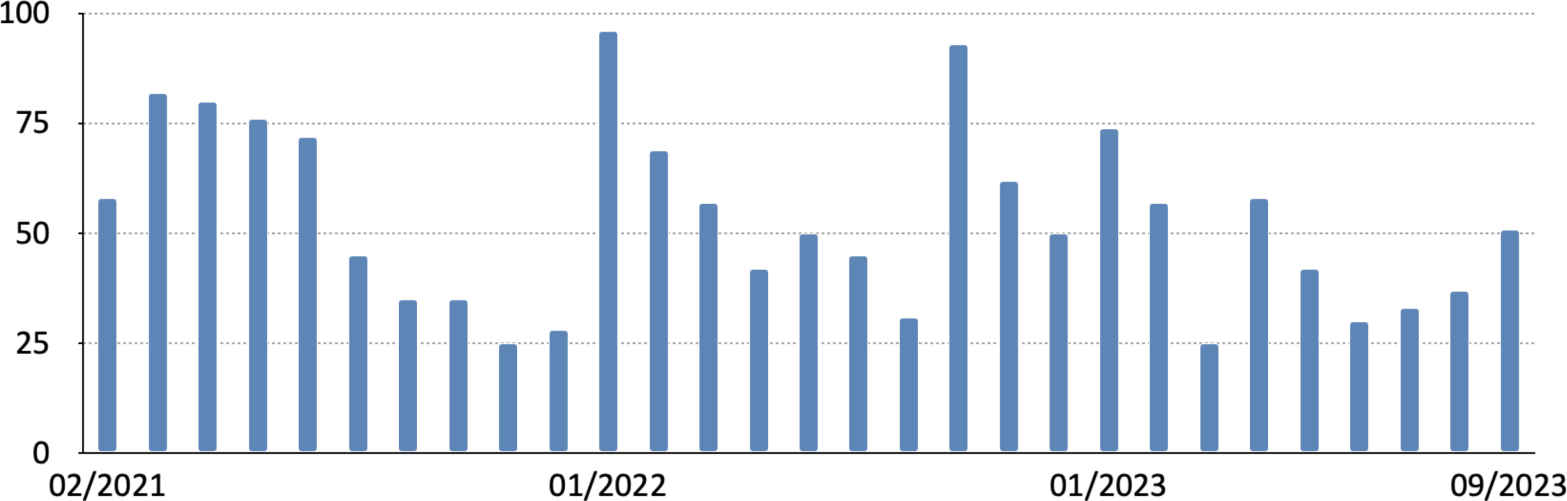
Number of participants per training session. Data from 23 sessions (Feb. 2021 to Sept. 2023)

**Figure 3:**
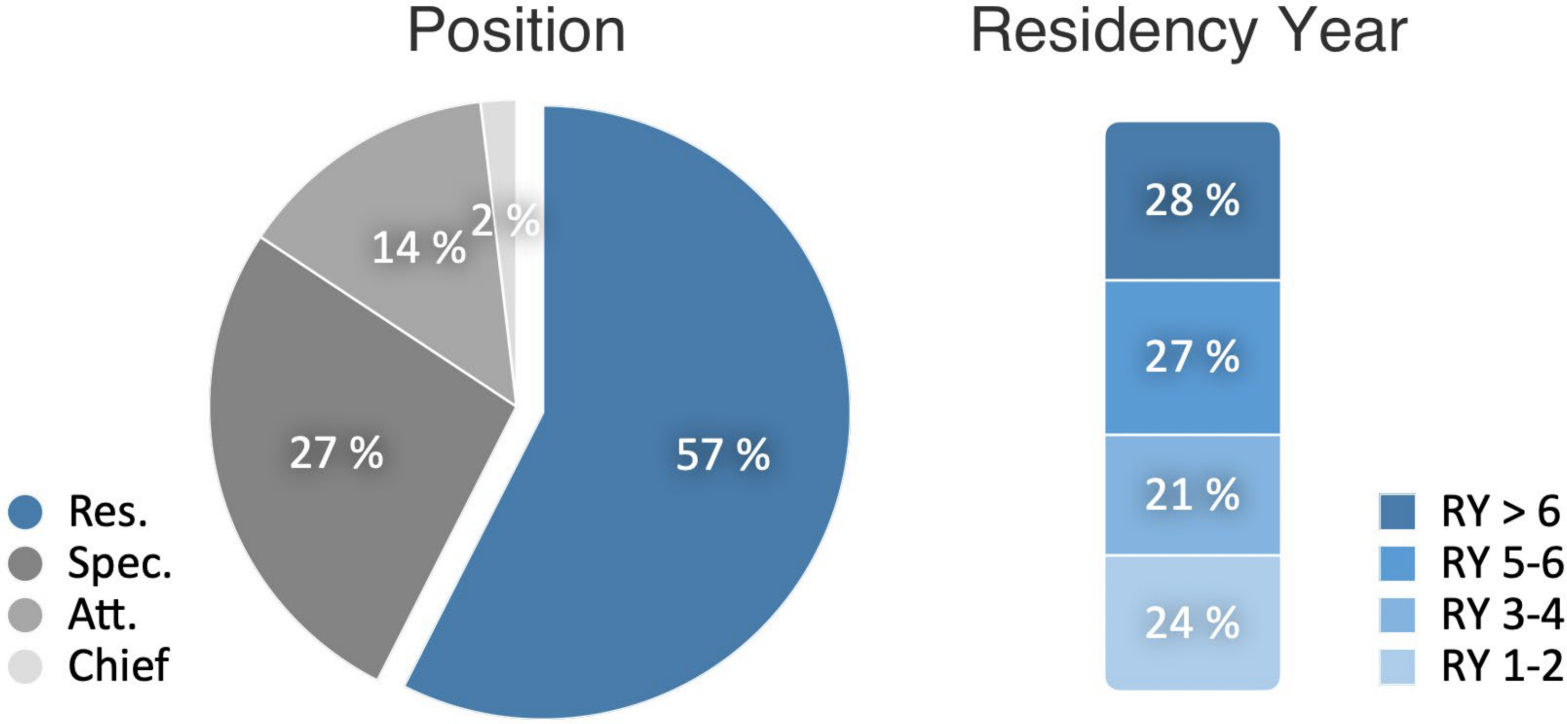
Job position of the participants as percentage of all participants, residency year as percentage of all participating residents. Evaluation data from 22 training sessions, n=347 (Res.=resident, Spec.=specialist, Att.=attending physician, Chief=chief of medicine. RY=residency year)

In 29 course sessions held between February 2021 and September 2023, a total of 1,538 individuals participated, with an average attendance of 53 attendees per session (M=53, min. 25, max. 96, SD=20). Participant numbers varied significantly depending on the date and topic (see table 1 (Tab. 1)), with fluctuations largely independent of the pandemic’s progression and the extent of social restrictions (see figure 2 (Fig. 2)). Notably, the originally targeted resident group constituted just over half of the participants, with a significant number of specialist physicians, senior hospital physicians, and chief hospital physicians also in attendance. Additionally, residents in their early training years (1-4) comprised less than half of the participating residents (see figure 3 (Fig. 3)). Based on the continuing education credit points applied for, it was evident that pediatric surgeons in private practice regularly attended KiWI sessions. On average, 16 participants (min. 4, max. 31) per course session applied for continuing education credit.

In the early sessions, 69% of participants indicated 5:30 p.m. as the preferred time, with only 3% favoring a 4:30 p.m. start. Participation primarily occurred via mobile devices, particularly smartphones. During sessions, most participants chose to keep their cameras turned off, yet there was a high willingness to utilize the chat function and respond to multiple-choice questions for interaction. Unfortunately, download counts for recordings from the DGKCH website could not be tracked for technical reasons. Fortunately, there were no issues in recruiting lecturers, who, in part, proactively suggested topics of their own initiative. All of these have so far been accepted.

Evaluation results for 22 course sessions are available (from Feb. 2021 to June 2021, and Jan. 2022 to August 2023). Participation in evaluations ranged from four to 43 individuals per session, yielding a response rate of 9% to 66%. Over time, there was a notable decrease in evaluation participation. A total of 363 datasets were analyzed, resulting in an overall response rate of 23.6%. Evaluation data analysis was conducted using Microsoft Excel^®^. Mean values per session were calculated based on the evaluation results, which were then averaged to obtain overall values (see table 2 (Tab. 2)). The selection of topics received high ratings (M=1.4), with no clear standout topic correlation. Participants felt actively engaged in the course, able to ask questions, and found case presentations consistently helpful to their learning. On average, case presentations were rated higher than lectures by presenters. The most notable discrepancies observed among individual sessions pertained to the participants’ perceived knowledge gain.

Open-ended comments praised the interactive design, practical relevance of case presentations, voluntary commitment of the organizational team, and session recording as a video podcast. Mixed feedback was received regarding desired main topics, with “traditional” pediatric surgery topics such as congenital deformities, aspects of pediatric traumatology, and practical instruction being most frequently requested.

## 4. Discussion

A tuition-free, monthly, post-graduate training program was introduced in February 2021 to provide advanced training in pediatric surgery for residents amidst the COVID-19 pandemic and the subsequent ban on in-person meetings. Through voluntary organization, the willingness of lecturers, and the utilization of existing technical resources, this series of training sessions was implemented with reasonable effort and without additional financial costs. Although post-graduate training should take place during working hours, KiWI sessions were scheduled based on participants’ preferred time outside of core working hours to accommodate their needs and maximize resident attendance. However, integrating theoretical training content into core working hours would be desirable for future iterations, although the decentralized nature of post-graduate surgical education poses challenges to structured training.

Attendance varied depending on the selected topic and lecturers but remained consistently high regardless of the pandemic’s course. Even after the end of social restrictions, a sustained level of interest underscored the value of online programs in post-graduate medical education. Each year around 200 people participate in AKIC, according to DGKCH. In 2022, 93 people participated in the review course *“Fit für den Facharzt”*, according to its organizer. For KiWI, an average of 53 people attended per session.

Although designed for pediatric surgery residents, specialist physicians, senior hospital physicians, and physicians outside of hospital settings also participated regularly. Even experienced participants were satisfied with the course. The comparatively lower assessments of the gains in knowledge may be attributed to the disparity between the targeted group and actual participants. Retaining ease of access for participation and ensuring close collaboration with residents in selecting topics would be recommended for upcoming KiWI sessions.

Due to limitations in available data, systematic analysis of evaluations based on participants' post-graduate education levels could not be conducted meaningfully. Detailed consideration could be given in the future when more evaluation results are available. However, direct feedback from residents during biannual assistants’ meetings provided valuable insights into their KiWI experience. The topics and the design of the training program were discussed individually in person.

The most commonly reported problems in connection with online education involve technical deficiencies, such as issues with connection or sound [18]. Implementing a mandatory, pre-scheduled technical check significantly enhanced the seamless delivery of the course. Session moderators supported presenters, managed surveys, and consolidated questions in the chat for efficient handling. Participants benefited from the integration of survey tools and the chat function within Zoom^®^, eliminating the need to switch between multiple programs.

For many online courses, the lack of opportunity for participants to interact and give feedback is described as problematic [19]. Activating methods were used to make KiWI interactive by design. Resident case presentations, which were highly regarded by participants, served as an effective teaching strategy to establish clinical references and enhance learning outcomes [15], [17]. Attention and active listening, which lead to better long-term recall, can be heightened by engaging the participants through multiple-choice questions [15], [20], [21]. Despite participants often having their cameras turned off, interaction between participants and presenters was facilitated through multiple-choice questions and the chat function, as is reflected by the evaluation results.

Conducting sessions as live event with subsequent availability in the form of a podcast, as described by various programs [22], appears advantageous. This format allows for real-time interaction during sessions and provides flexibility for DGKCH members to access recordings at their convenience. Moreover, the collected recordings can be used to prepare for the pediatric surgery board examination.

As invitations were primarily distributed through DGKCH channels, they predominantly reached residents and physician specialists who were further along in their post-graduate training, as they are more likely to be members of the society. This raises the question of how to better reach residents in the early stages of their specialist training using this format. To address this, efforts were made to increase awareness through social media dissemination and targeted outreach to younger colleagues in locations where post-graduate training occurs. Moving forward, it is imperative to specifically inform individuals responsible for resident training about this program to ensure broader reach and inclusivity across all stages of residency.

As a limitation, it must be noted that the training program is designed specifically for German-speakers and tailored to a relatively small subject area covering a wide spectrum. Since residents in other areas may have different needs when it comes to post-graduate education, we recommend conducting a needs assessment that includes the target group prior to implementation.

Post-graduate surgical training must impart both theoretical knowledge and practical skills. Competency-based training for students in the fifth year of undergraduate medical study have been successfully implemented in a telemedicine-based format [23]. Additionally, tele-mentoring has been shown to facilitate the acquisition of surgical skills, such as laparoscopic knot-tying techniques [24]. Looking ahead, these approaches could be extended to post-graduate medical education. There are diverse opportunities to enhance decentralized programs that facilitate and support on-site post-graduate training within hospitals. By leveraging telemedicine and tele-mentoring technologies, educators can offer valuable training experiences that bridge geographical barriers and provide enhanced learning opportunities for surgical trainees.

## 5. Conclusion

KiWI, an online training program for pediatric surgery residents, was successfully implemented with a reasonable amount of human and financial resources. This online training program can help pediatric surgery residents to improve their theoretical and practical knowledge and, thus, support and complement the post-graduate training at their hospitals. The advantages of decentralized, competency-based training can be realized through online seminars using interactive, case-based teaching methods. Online education is a resource-efficient addition to post-graduate training in pediatric surgery and should, in the authors’ opinion, become a permanent component of post-graduate curricula to take on the challenges of the evolving healthcare system and changing needs of residents.

## Authors’ ORCIDs

- Richard Gnatzy: [0009-0003-1308-5568]
- Benjamin Schwab-Eckhart: [0009-0006-9299-9254]
- Annika Brunner: [0009-0008-2799-9789]
- Sabine Drossard: [0000-0002-3442-4851]

## Acknowledgements

We extend our thanks to all of the presenters who, with great commitment, supported this project and contributed to its success thus far. We also thank Robert Lauch, who played a significant role in the project as part of the organizational team. Our thanks also go to Prof. Dr. Udo Rolle and the Deutsche Gesellschaft für Kinderchirurgie for their support in realizing this project.

## Competing interests

The authors declare that they have no competing interests. 

## Figures and Tables

**Table 1:**
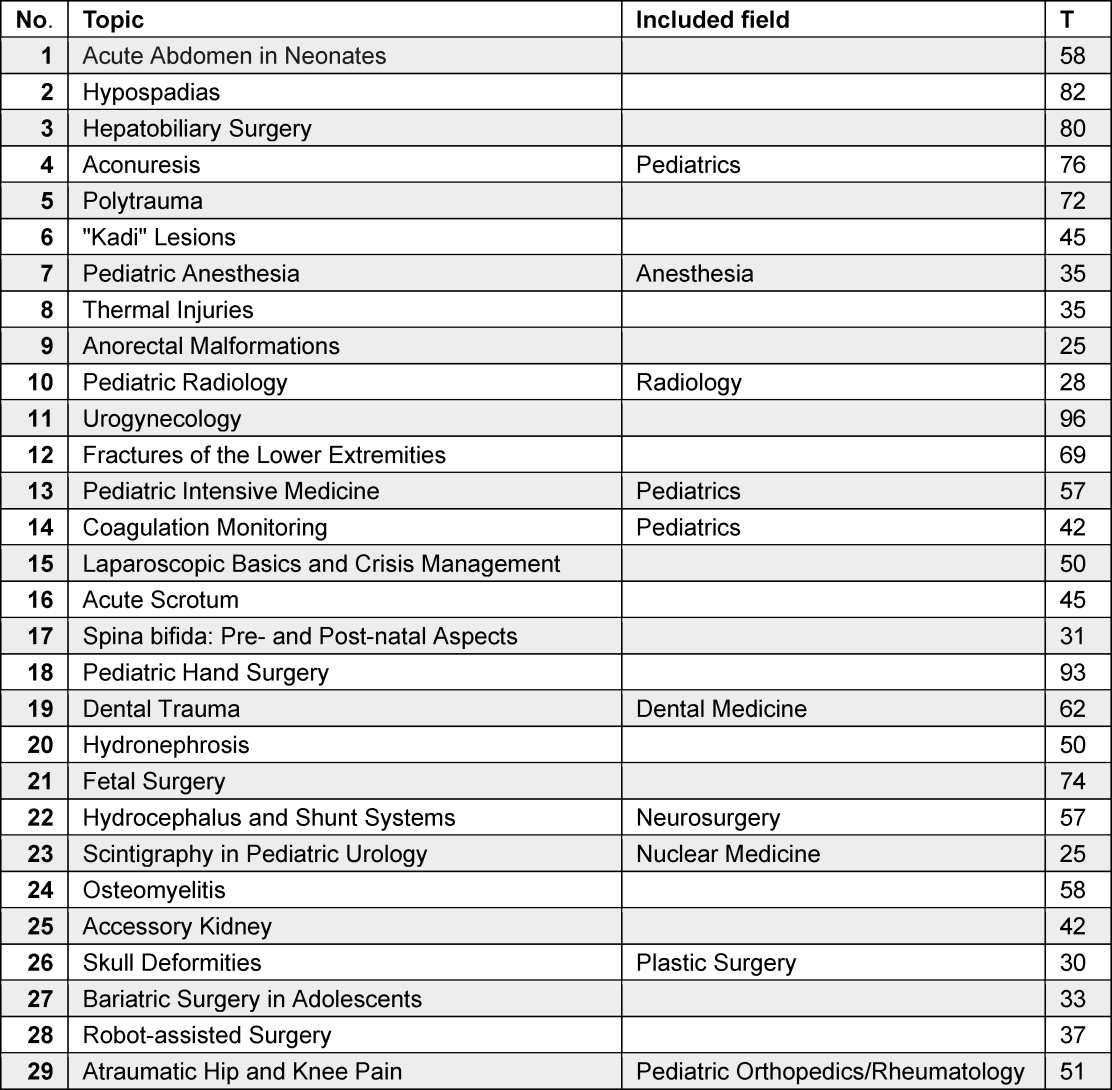
List of topics and level of attendance (T=number of participants)

**Table 2:**
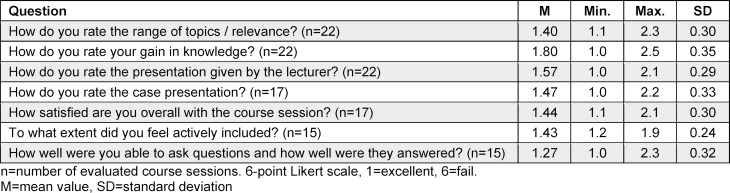
Evaluation results
